# Dynamic multivoxel‐localized ^31^P MRS during plantar flexion exercise with variable knee angle

**DOI:** 10.1002/nbm.3905

**Published:** 2018-03-26

**Authors:** Fabian Niess, Georg B. Fiedler, Albrecht I. Schmid, Elmar Laistler, Roberta Frass‐Kriegl, Michael Wolzt, Ewald Moser, Martin Meyerspeer

**Affiliations:** ^1^ Center for Medical Physics and Biomedical Engineering Medical University of Vienna Austria; ^2^ Highfield MR Center Medical University of Vienna Austria; ^3^ Department of Clinical Pharmacology Medical University of Vienna Austria

**Keywords:** exercise, knee angle, multi‐voxel, muscle, phosphorus MRS/MRSI, spectroscopic localization

## Abstract

Exercise studies investigating the metabolic response of calf muscles using ^31^P MRS are usually performed with a single knee angle. However, during natural movement, the distribution of workload between the main contributors to force, gastrocnemius and soleus is influenced by the knee angle. Hence, it is of interest to measure the respective metabolic response of these muscles to exercise as a function of knee angle using localized spectroscopy.

Time‐resolved multivoxel ^31^P MRS at 7 T was performed simultaneously in gastrocnemius medialis and soleus during rest, plantar flexion exercise and recovery in 12 healthy volunteers. This experiment was conducted with four different knee angles.

PCr depletions correlated negatively with knee angle in gastrocnemius medialis, decreasing from 79±14 % (extended leg) to 35±23 %(∼40°), and positively in soleus, increasing from 20±21 % to 36±25 %; differences were significant. Linear correlations were found between knee angle and end‐exercise PCr depletions in gastrocnemius medialis (R ^2^=0.8) and soleus (R ^2^=0.53). PCr recovery times and end‐exercise pH changes that correlated with PCr depletion were consistent with the literature in gastrocnemius medialis and differences between knee angles were significant. These effects were less pronounced in soleus and not significant for comparable PCr depletions. Maximum oxidative capacity calculated for all knee angles was in excellent agreement with the literature and showed no significant changes between different knee angles.

In conclusion, these findings confirm that plantar flexion exercise with a straight leg is a suitable paradigm, when data are acquired from gastrocnemius only (using either localized MRS or small surface coils), and that activation of soleus requires the knee to be flexed. The present study comprises a systematic investigation of the effects of the knee angle on metabolic parameters, measured with dynamic multivoxel ^31^P MRS during muscle exercise and recovery, and the findings should be used in future study design.

**Abbreviations used**
ANOVA: analysis of variance
HSD: Tukey's honestly significant difference
MR: magnetic resonance
MRS: magnetic resonance spectroscopy
SD: standard deviation
SNR: signal‐to‐noise ratio
SVS: single‐voxel spectroscopy.

## 1 INTRODUCTION

Exercising muscle tissue has been studied for several decades using magnetic resonance spectroscopy (MRS).(1, 2) In particular, time‐resolved ^31^P MRS is a valuable non‐invasive tool to investigate the metabolic response of exercising muscle in humans. Physiologically relevant parameters such as pH, PCr recovery time constant *τ*_PCr_ and maximum oxidative capacity *Q*_max_(3, 4) can be calculated from time courses of phosphocreatine and inorganic phosphate.^5^

A challenge with dynamic studies of muscle tissue is to achieve the temporal resolution to follow the course of the target metabolites, while localizing the origin of the signal to active muscle only and maintaining sufficient signal‐to‐noise ratio (SNR). Several exercise‐recovery studies differentiating signals from gastrocnemius and soleus, which are the main contributors to force in plantar flexion, have been published using either localized MRS(6, 7, 8, 9) or MRI.(10, 11, 12, 13, 14) Both muscles act on the Achilles tendon and are attached to different positions on the proximal end. Soleus is connected to the tibia and gastrocnemius lateralis and medialis originate at the femur; therefore changes of the knee angle affect these muscles to a different extent.(15, 16) Additionally, the fibre‐type composition differs between these calf muscles: soleus is predominantly composed of slow twitch fibres (80 %), while a more even distribution of slow and fast twitch fibres has been reported in gastrocnemius.(17, 18) Because of these biomechanical and anatomical differences, the knee angle is a crucial parameter determining the distribution of workload between calf muscles during plantar flexion exercise.^19^ Previous studies have shown that soleus is more activated with a flexed knee, while gastrocnemius contributes more with an extended knee. Price et al^16^ used MRI and surface electromyography to study the pre‐ versus post‐exercise changes of *T*_2_ and electromyography activity in two groups of 12 subjects exercising at three different knee angles. Valkovic et al^20^ acquired dynamic spiral spectroscopic ^31^P magnetic resonance (MR) images in five volunteers performing plantar flexion with two different knee angles. To our knowledge, measurements of dynamic ^31^P MR spectra of exercising calf muscle with a variation of knee angle have not been made so far in more than two positions. However, this allows a more detailed quantification of the influence of knee angle on the distribution of the workload between calf muscles contributing to plantar flexion in a natural movement pattern.

Recently, a semi‐localization by adiabatic selective refocusing (LASER) sequence(21, 22) has been developed, which is capable of acquiring spectra from multiple independent volumes in an interleaved fashion.^23^ This technique provides the same temporal resolution as the original single‐voxel spectroscopy (SVS) sequence, but acquires data from two voxels. It is therefore a suitable technique to measure the metabolic response of two different muscles dynamically during a single exercise‐recovery experiment.

This work investigates the impact of the knee angle on the distribution of workload between gastrocnemius and soleus during plantar flexion^9^ by executing this exercise at four different knee angles and acquiring time‐resolved ^31^P spectra at 7 T, using a multivoxel semi‐LASER sequence.

## 2 EXPERIMENTS

Magnetic resonance spectroscopy data from gastrocnemius medialis and soleus of healthy volunteers were acquired simultaneously during rest, exercise and recovery, repeatedly for each knee angle. 12 subjects (8 males and 4 females, age 26.4 ± 4.9 years, body mass index 22.1 ± 1.7 kg/m^2^) participated in the study after having declared written informed consent to the protocol, which is in accordance with the guidelines of the local ethics committee and the latest version of the declaration of Helsinki. All subjects were recreationally physically active.

The measurement protocol consisted of 1 min rest, 3 min exercise and 5 min recovery using a custom‐built pneumatic pedal ergometer (similar to that described in Meyerspeer et al^24^) for plantar flexion during the MR measurements. The exercise intensity was standardized for all measurements by inflating the pneumatic system to 0.4 bar, using a manual pump. The subjects were instructed to time the plantar flexion exercise so that tissue motion during data acquisition was minimized, with a single pedal push between the MRS acquisitions, every 3 s. The force was measured continuously during exercise, using sensors on the ergometer and recorded by an external computer in the control room.

A multichannel (two channels ^1^H, three channels ^31^P) surface coil array, which was shaped to a half cylinder (*d*=14 cm,*l*=10 cm) to match the anatomy of a human calf,^25^ was used for RF transmit and receive in a 7 T Magnetom whole‐body MR system (Siemens Healthcare, Erlangen, Germany). The coil was placed on a support with adjustable angle (in steps of 5°), which was positioned on plastic spacers to adapt the height. Each subject performed a total of four exercise‐recovery bouts, each with a different angle of the support (0° , 5° , 10° , 15°), designed to flex the knee by angles between 10° and 40°. The order of the measured knee angles was randomized between subjects, to rule out the possible effects of insufficient recovery from exercise. With this range of inclinations, the space available with the configuration used in the magnet bore was exploited to its maximum. The corresponding knee angle was measured with a protractor, using the lines from the knee to the hip (with the greater trochanter as a marker) and from the knee to the ankle as reference. This measurement was done repeatedly (five measurements per angle) in five representative subjects. The angle of the pedal was adapted to the posture of the leg, to always achieve a 90°  angle between the foot and the lower leg in the neutral position.

The acquisition time of ^31^P MRS data for each knee angle was 9 min, followed by a minimum pause of 15 min for angle adjustment, which required retraction of the patient bed from the scanner and repositioning of the subject. Consequently, the time between the end of an exercise bout and the start of the subsequent exercise was at least 21 min. During this period the muscle was at rest and PCr, Pi and the pH were allowed to recover to basal levels. Ten subjects were scanned in two sessions on different days, studying two knee angles per session, while it was possible to measure two subjects in a minimum total measurement time of 90 min, each, consisting of less than 10 min for initial preparation in the scanner room, 4 x 9 min acquisition time and 3 x 15 min for subject repositioning and angle adjustments.

A dynamic localized multivoxel ^31^P MRS sequence with adiabatic refocusing pulses (semi‐LASER)^23^ was used to select anatomy‐matched voxels in the muscles of interest, gastrocnemius medialis and soleus. The voxels were carefully positioned, taking into account the angle of the calf, with the help of a localizer image and 25 transversal gradient‐echo MRI slices, which were acquired at each angle. The average ^31^P MRS voxel sizes were VOI_gastroc._=55 ± 12 cm^3^ and VOI_soleus_=51 ± 9 cm^3^, respectively. The voxels in gastrocnemius medialis and soleus were measured interleaved with a delay of 3 s (i.e. with a total repetition time of 6 s, corresponding to the effective recovery time for *T*_1_ relaxation with this acquisition scheme^23^) and an echo time of *T*_E_=29 ms for each voxel. Calibration scans were performed in each subject before the exercise‐recovery experiments to adjust the RF transmit voltages, using the multivoxel ^31^P MR sequence. The excitation pulse duration for both voxels was 2.6 ms and the adiabatic refocusing pulse duration was 4.6 ms. It is important to ensure that excitation slices of the voxels do not overlap, to avoid unwanted mutual saturation. In contrast, an overlap of the refocusing slices is acceptable, as the corresponding saturation is negligible with the set‐up used.^23^ The voxel in gastrocnemius is always located closer to a surface coil placed below the calf than the one in soleus and therefore a lower *B*_1_ amplitude is required for refocusing. However, since refocusing planes mutually overlap between voxels in gastrocnemius and soleus, *B*_1_ amplitudes of both voxels need to be equal, to ensure full refocusing in soleus, as described by Niess et al. 23 The shim volume was set up as small as possible, but sufficiently large to contain both voxels at once.

NMR raw data were extracted and processed using in‐house developed Python scripts. Phosphocreatine (PCr) and inorganic phosphate (Pi) resonances were quantified using the fitting routine AMARES 26 in jMRUI.^27^ The pH value was calculated from the chemical shift of PCr and inorganic phosphate for each pair of effective averaged acquisitions using jMRUI.^28^ PCr depletions (*d*_PCr_ ) were calculated by normalizing every acquisition to the median of the last minute of recovery of the data set. Values for end‐exercise *d*_PCr_ are given as the mean and standard deviation (SD) of the last five acquisitions during exercise. Recovery time constants *τ*_PCr_ were fitted mono‐exponentially to the PCr recovery time course. Maximum oxidative capacity (*Q*_max_) was calculated from *d*_PCr_ , *τ*_PCr_ and pH using an ADP‐control model, as described in Kemp et al^3^ and Fiedler et al^4^ and using common assumptions mentioned by Kemp et al.^5^ The SNR was calculated from the fully relaxed first spectrum acquired at the beginning of each time series, using the amplitude of the PCr signal and the SD of noise measured 12 ppm off‐center across one eighth of the total bandwidth.

Statistical tests were performed on the results of *d*_PCr_ , end‐exercise pH (pH_ee_), *τ*_PCr_ and *Q*_max_ acquired for all knee angles. One‐way analysis of variance (ANOVA) with a post‐hoc analysis using Tukey's honestly significant difference (HSD) was used where applicable, i.e. when data followed a normal distribution; otherwise Friedman's test combined with Nemenyi's post‐hoc test was used.

## 3 RESULTS

The SNR of PCr at rest did not change significantly between knee angles and was 120 ± 24 in gastrocnemius medialis and 42 ± 12 in soleus on average across all subjects (mean ± SD). The linewidths, quantified throughout the experiment, were 7.8 ± 1.2 Hz in gastrocnemius medialis and 8.3 ± 1.5 Hz in soleus, which is consistent with previously published data.^23^ The average knee angles measured in each position were 14°, 20°, 33° and 38°; the reproducibility across five repeated measurements was within 2°(SD). The average force that was applied on the pedal during exercise was 249 ± 60 N. The intrasubject variability between knee angles was 8 % (SD) on average. The corresponding power output was 6.2 ± 3.4 W, with a variation of 14 %. In Table 1, the group averages of end‐exercise PCr depletion, end‐exercise pH, PCr recovery time constant, linewidths and SNR are reported for each knee angle and muscle (gastrocnemius medialis and soleus).

**Table 1:** End‐exercise phosphocreatine depletion (d_PCr_), end‐exercise pH (pH_ee_), calculated recovery time constant (τ_PCr_), linewidth (lw) and signal‐to‐noise ratio (SNR) are given for each knee angle and muscle, i.e. gastrocnemius medialis (GM) and soleus (SOL), as mean ± SD over all subjects, respectively. Linedwidth and SNR were averaged throughout the experiment

knee angle	*d*_PCr_ GM [%]	**pH_ee_ GM**	***τ*_PCr_ GM [s]**	lw GM [Hz]	SNR
14°	79 ± 14	6.77 ± 0.12	61 ± 15	8.2 ± 3.2	122 ± 39
20°	68 ± 16	6.85 ± 0.13	47 ± 10	7.7 ± 2.3	118 ± 23
33°	47 ± 23^*a*^	6.97± 0.10^*a*^	49±23	7.1 ± 2.0	126 ± 29
38°	35 ± 23^*a*,*b*^	6.99 ± 0.12^*a*,*b*^	39 ± 17^*a*^	8.0 ± 3.0	113 ± 34
**knee angle**	***d*_PCr_ SOL [%]**	**pH_ee_ SOL**	***τ*_PCr_ SOL [s]**	**lw SOL [Hz]**	**SNR**
14°	20 ± 21	7.03±0.04	29±15	7.5 ± 2.4	46 ± 16
20°	20 ± 18	7.04 ± 0.03	24 ± 10	8.1 ± 2.2	40 ± 12
33°	30 ± 22	7.03 ± 0.05	38 ± 13	8.3 ± 2.4	43 ± 11
38°	36 ± 24^*b*^	7.03 ± 0.05	42 ± 35	9.2 ± 2.5	41 ± 16

^*a*^Significantly different from 14°(*p*<0.05).

^*b*^Significantly different from 20°(*p*<0.05).

**Figure 1:**
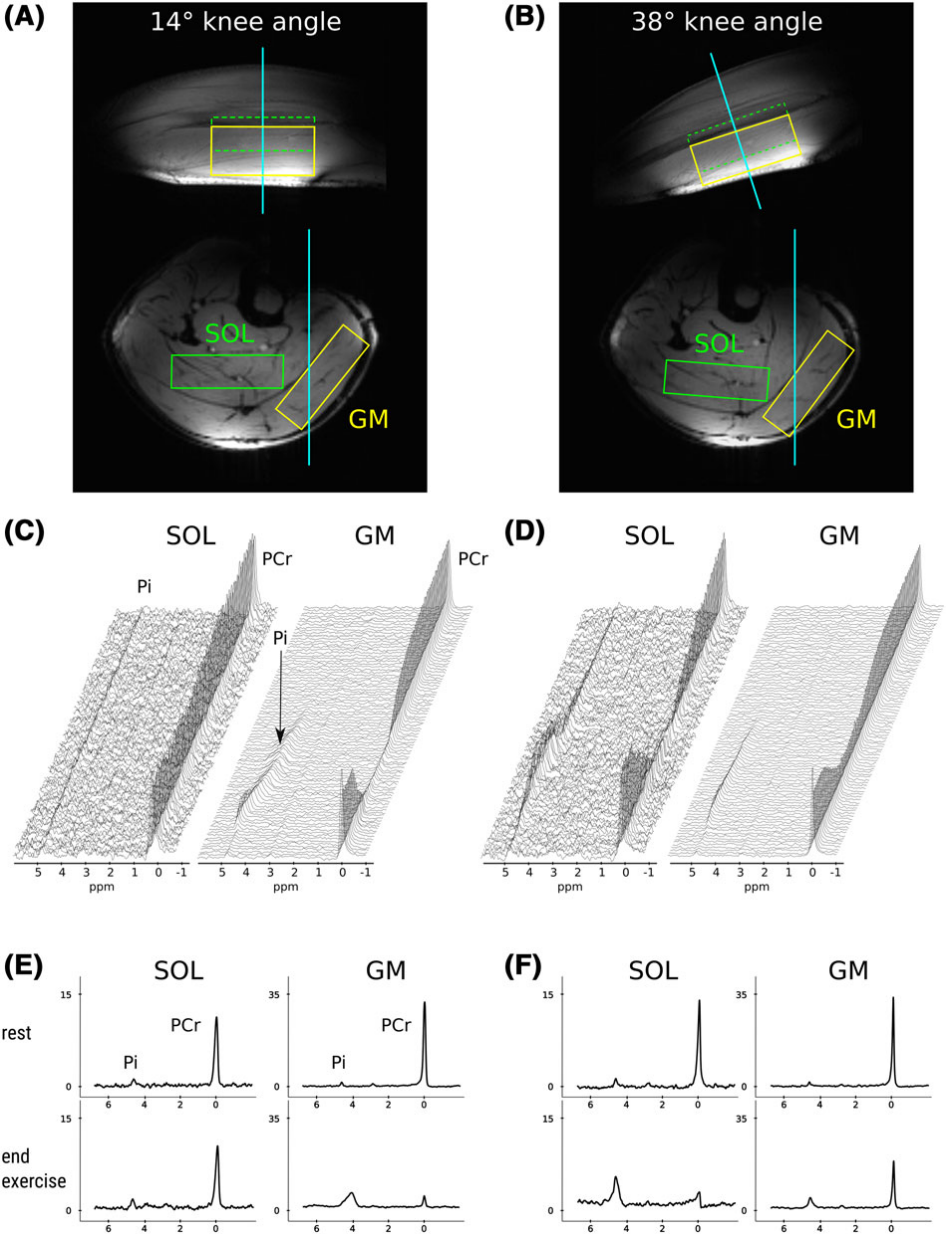
Sagittal and transversal gradient‐echo images of the calf with (A) a near‐straight leg and (B) 38° knee angle. Typical voxel positions for localized ^31^P–MRS of soleus (SOL, green) and gastrocnemius medialis (GM, yellow) are shown. Time series of spectra acquired at rest and during exercise and recovery with knee angles of (C) 14° and (D) 38° are shown for GM and SOL with a time resolution of 6 s. Corresponding ^31^P spectra averaged over 30 seconds (five spectra) of rest (top) and end of exercise (bottom) are shown in (E) and (F)

To illustrate the placement of ^31^P semi‐LASER volumes of interest (VOIs) in the calf muscles at different knee angles, gradient‐echo images of one representative subject are shown in Figure 1A and B. Spectra acquired with a time resolution of 6 s during rest, exercise and recovery with 14° and 38° knee angles are shown as time series in Figure 1C and D. Corresponding averaged spectra (*n*=5) of the last 30 seconds of rest and end‐exercise are shown in Figure 1E and F.

**Figure 2:**
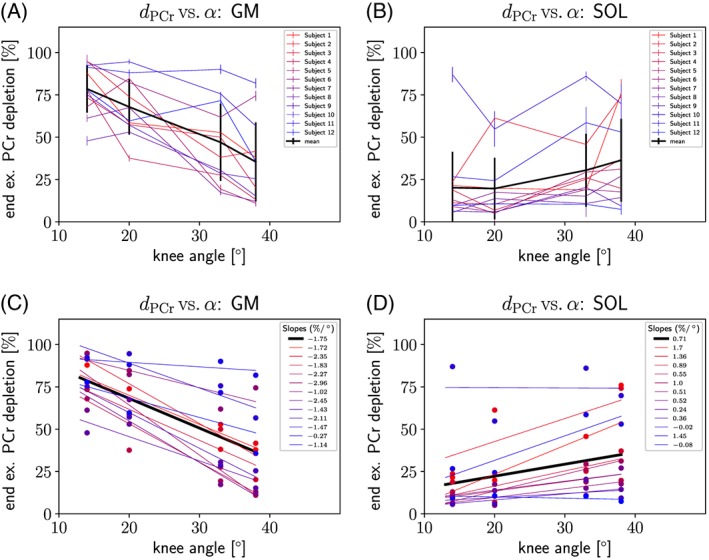
End‐exercise phosphocreatine depletion (d_PCr_) versus knee angle given for (A) gastrocnemius medialis and (B) soleus of each subject, with the overall mean (black line). Linear regression calculated for each subject is plotted as colored lines for (C) gastrocnemius medialis and (D) soleus, with their respective slopes shown in the legend

End‐exercise PCr depletion is plotted versus knee angle for gastrocnemius medialis in Figure 2A and for soleus in Figure 2B, for individual subjects (colored symbols) and as a group average (black). Linear regression was calculated for end‐exercise PCr depletion averaged across subjects at each knee angle (black line) and additionally for each subject separately (colored lines); see Figure 2C and D.

In gastrocnemius medialis, *d*_PCr_ correlated negatively with the knee angle and values showed significant differences (*p*<0.05); see Table 1. In contrast, the correlation found in soleus was positive and end‐exercise PCr depletion changed significantly with the angle of the knee.

In both calf muscles studied, a linear relationship was found between the knee angle and *d*_PCr_. The coefficient of determination of the linear regression was *R* ^2^=0.80 ± 0.16 in gastrocnemius and *R* ^2^=0.53 ± 0.24 in soleus, given as mean ± standard deviation (SD) across all subjects. Individual and averaged results (*R* ^2^ and slopes) of all subjects are shown in Table 2. The slopes of all linear regression lines for individual subjects were negative in gastrocnemius and the majority of the slopes were positive in soleus. Only two subjects showed negative but very small slopes in soleus (−0.02 and −0.08), while one of these subjects showed very high (74 ± 15 *%*) and very low (9.5 ± 1.5 *%*) end‐exercise PCr depletion on average over all knee angles.

**Table 2:** Coefficients of determination (R ^2^) and slopes calculated using linear regression between end‐exercise PCr depletion and the knee angle (mean ± SD) for each subject. Parameters are given for gastrocnemius medialis (GM) and soleus (SOL)

Subject [No.]	*R* ^2^ GM	*R* ^2^ SOL	slope GM [*%*/° ]	slope SOL [*%*/° ]
1	0.84	0.46	−1.73	1.70
2	0.94	0.49	−2.34	1.36
3	0.84	0.62	−1.79	0.89
4	0.84	0.68	−2.28	0.55
5	0.83	0.77	−2.95	1.00
6	0.69	0.77	−1.02	0.51
7	0.90	0.64	−2.48	0.52
8	0.86	0.44	−1.45	0.24
9	0.98	0.35	−2.12	0.36
10	0.86	0.00	−1.47	−0.02
11	0.50	0.84	−0.27	1.45
12	0.47	0.32	−1.07	−0.08
mean	0.80 ± 0.16	0.53 ± 0.24	−1.75 ± 0.74	0.71 ± 0.58

**Figure 3:**
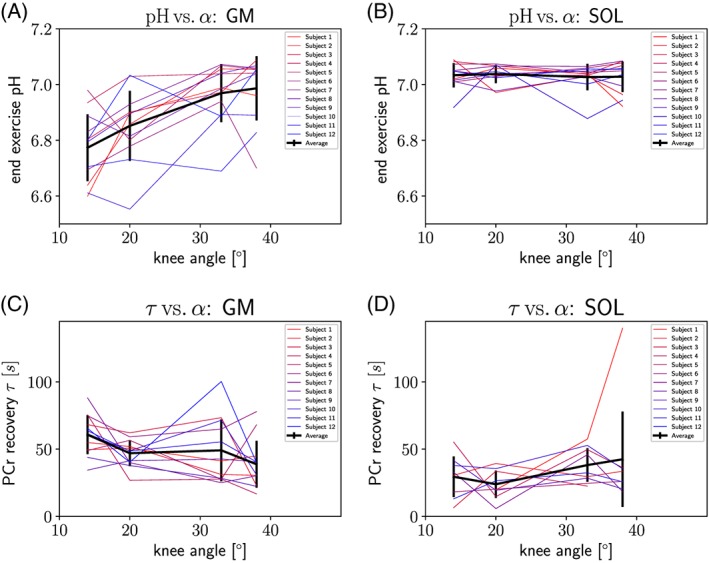
End‐exercise pH versus knee angle given for (A) gastrocnemius medialis and (B) soleus, individually for each subject (colored) and averaged (black). PCr recovery time constant τ_PCr_ computed from a mono‐exponential fit is plotted versus the knee angle for (C) gastrocnemius medialis and (D) soleus, individually (colored) and averaged (black)

Calculated end‐exercise pH in gastrocnemius medialis increased significantly with the angle of the knee (*p*<0.05), from 6.77 ± 0.12 to 6.99 ± 0.12, as shown in Table 1. In soleus, pH_ee_ was independent of the knee angle when regarding group averages, as shown in Figure 3 and Table 1.

The PCr recovery time constants *τ*_PCr_ increased significantly along with the acidification in gastrocnemius medialis (*p*<0.05; see Figure 3C and Table 1). The increase found in soleus (see Figure 3D) did not reach statistical significance. Data points for *τ*_PCr_ were excluded from the analysis if the standard deviation exceeded 30 s (presumably indicating impaired reliability of the PCr recovery fit, related to insufficient PCr depletion). Data points of gastrocnemius were not affected, but 13 out of 48 data points from soleus were excluded.

**Figure 4:**
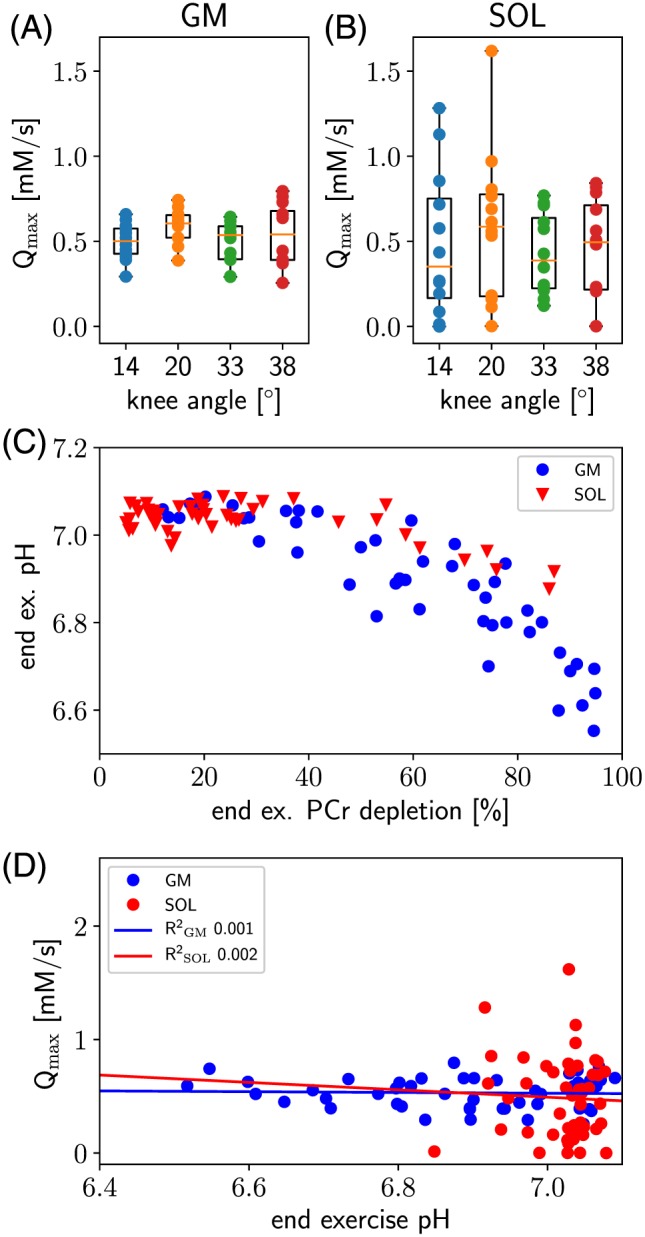
The maximum oxidative capacity calculated for all knee angles (14°, 20°, 33°, 38°) illustrated as a box and whiskers plot for (A) gastrocnemius medialis and (B) soleus. The relationship between the pH at the end of exercise and the corresponding amount of PCr depletion is shown in (C). Correlation between end‐exercise pH and maximum oxidative capacity is shown in (D)

Maximum oxidative capacity *Q*_max_ was 0.53 ± 0.13 mM/s on average in gastrocnemius medialis and 0.49 ± 0.36 mM/s in soleus (0.45 ± 0.25 mM/s when averaging data were acquired with 33° and 38° knee angle only). Individual *Q*_max_ data points, together with a box and whiskers plot, are shown for all knee angles in Figure 4A and B.

A one‐way ANOVA test in both calf muscles studied showed that *Q*_max_ did not change significantly with different knee angles.

A significant pH drop was observed above a threshold of approximately 60*%* PCr depletion in gastrocnemius medialis, as previously reported by Fiedler et al.^4^ Figure 4C shows the relationship between end‐exercise PCr depletion and end‐exercise pH for both muscle groups. Only a few data points from soleus showed depletions above 60*%* (*n*=6), which was associated with an average pH of 6.93 ± 0.03. When comparing these data points with data from gastrocnemius falling in the same range of PCr depletion, i.e. between 60*%* and 87*%*(*n*=15), a significantly lower end‐exercise pH 6.85 ± 0.07 was found for gastrocnemius (*p*<0.05, Wilcoxon rank‐sum test). The corresponding maximum oxidative capacities were not significantly different with *Q*_max_=0.47 ± 0.12 mM/s (gastrocnemius) and *Q*_max_=0.60 ± 0.22 mM/s(soleus).

The correlation between *Q*_max_ values and end‐exercise pH of gastrocnemius medialis and soleus is shown in Figure 4D.

## 4 DISCUSSION

A relation between knee angle and the recruitment pattern of gastrocnemius and soleus is expected according to anatomy and has been explored with ^1^H MRI^16^ and ^31^P MRS.^20^ The purpose of this study was to investigate the effect of knee angle on metabolic changes in those muscles in further detail, in order to establish a more comprehensive model by acquiring localized ^31^P MRS data in exercising calf muscles with a simple and time‐efficient method with four different knee angles.

The metabolic parameters measured with localized dynamic ^31^P MRS, which are linked to muscle activation (i.e. PCr depletion, pH_ee_ and *τ*_PCr_), showed a strong dependence on the angle of the knee during plantar flexion.

In gastrocnemius, a strong correlation was found between the knee angle and the end‐exercise PCr depletion. Results showed a linear behaviour, with a decreasing metabolic response with increasing knee angle. PCr depletion, end‐exercise pH and PCr recovery time were significantly different between measurements. The observed relationship between strong muscle activation, evidenced by PCr depletion, acidification and a concurrent increase of *τ*_PCr_, is consistent with previous results.(4, 22, 29, 30)

In soleus, moderate to strong linear correlations between knee angle and PCr depletion were observed, with the opposite sign to those in gastrocnemius, i.e. an increasing knee angle was associated with increasing PCr depletion in most subjects. The effect of the knee angle on the derived parameters was, however, less pronounced in soleus than in gastrocnemius, i.e. PCr depletion in soleus was significant, but did not reach the levels of depletion in gastrocnemius, and pH_ee_ remained neutral or indicated only mild acidification. The variability of PCr depletion was more pronounced in soleus than in gastrocnemius, especially in two subjects who recruited soleus to the same extent for all knee angles, resulting in similar slopes close to zero (−0.02 and −0.08), but, interestingly, showed significantly different PCr depletion (74 ± 15 *%*) and (9.5 ± 1.5 *%*). It is very unlikely that this observation is due to a motion artifact; it may reflect apparent differences in types of training and individual natural movement patterns between subjects, which could impact the recruitment of soleus during plantar flexion exercise. Furthermore, this might be a consequence of using the same workload for all volunteers, although the majority of subjects showed comparable changes of muscle recruitment between different knee angles. However, the intrasubject variability of the measured force between knee angles was 8 %, which allows for investigation of muscle groups contributing differently with a variable knee angle at a constant force output. Despite the inter‐subject variability of end‐exercise PCr depletion (in both muscles), the coefficients of determination of the linear fits and the slopes were consistent and similar within each muscle, which supports the hypothesis that the knee angle has a strong role in determining the distribution of workload between the two calf muscles studied.

The PCr recovery time constants *τ*_PCr_ quantified in both muscles were in good agreement with the literature.(4, 11) When using localized spectroscopy, only tissue from a single muscle contributes to the signal and therefore a mono‐exponential fitting routine during PCr recovery was used. Fitting a bioexponential model to our data was tested and resulted in equivalent results, i.e. the PCr recovery times of both compartments were identical (differences smaller than 1 %). When PCr depletion was low, biexponential fit results were not reliable.

Knee angulation modulates the level of muscle activation, but did not influence the calculated mitochondrial capacity *Q*_max_ derived from the ADP model^3^ of both muscle groups. This demonstrates the robustness of the applied method. Calculated *Q*_max_ values presented here are in excellent agreement with the publication of Fiedler et al^4^ which contains results from gastrocnemius medialis and soleus measured in another cohort of subjects, and also with the work of Schmid et al^14^ reporting results from gastrocnemius medialis only. Values of *Q*_max_ from gastrocnemius were reliable for all knee angles, as the PCr depletion was sufficient in most of the subjects shown in Figure 4A. *Q*_max_ results from soleus showed a higher variability, especially with the knee flexed by 14° or 20°, since end‐exercise PCr depletion in the majority of subjects was too low in soleus to obtain highly accurate results with these knee angles. For 33° and 38°, the *Q*_max_ obtained was more robust and more similar to results of gastrocnemius medialis on average; see Figure 4B. In contrast to the publication of Fiedler et al^4^ neither gastrocnemius nor soleus showed a correlation between $Q_{max}$ and end‐exercise pH; see Figure 4D. This could be explained by the fact that we acquired three times more data points in total, with four acquisitions per subject, over a wider range of stimulation intensities per muscle. Significant differences of muscle activation between knee angles were found, while *Q*_max_ values did not change significantly. This is consistent with the notion that maximum oxidative capacity is an intrinsic parameter to the muscle tissue and should be independent of the degree of stimulation, provided there is sufficient oxygen supply.

The results of all four knee angles did not show any sign that the time for the muscles to return to the basal state would have been too short and that possible residual effects on enzymatic activity, glycogen repletion, water content or vasoreactivity were not taken into account in this study.

To maintain a coherent exercise protocol, the same force was chosen for all knee angles (similar to Price et al^16^). This resulted in an exercise response for gastrocnemius that can be explained consistently as decreasing recruitment with a bent knee. Gastrocnemius is a bi‐articular muscle, i.e. it crosses two joints in series and is therefore shortened in length with a flexed knee, while soleus is attached to the tibia and does not change in length by knee angulation.(15, 16) Muscle shortening has been linked to a decrease in the number of available sites for cross‐bridge formation,^19^ which influences a muscle's force output^31^ and hence energy cost.^32^ Furthermore, the relation between sarcomere length and force output depends on the type of muscle.^31^ This may contribute to the explanation of why PCr depletion in soleus did not increase to the same extent as the corresponding decrease in gastrocnemius. The slope of the linear regression of depletion versus angle was smaller in soleus and, consequently, maximum depletions typically reached in soleus were smaller than the strong depletions found in gastrocnemius medialis. A further possible explanation for these findings is that soleus differs from gastrocnemius in fibre‐type composition^17^ and cross‐section area, therefore it could be capable of producing higher maximum force output or it might show a different metabolic reaction to an equivalent workload. However, changing the ergometer pedal's recoil force with angle would render the protocol inconsistent (particularly for the measurement in gastrocnemius). Moreover, additional measurements with increased force and a flexed knee were attempted, but did not generate similar depletion to that in gastrocnemius medialis or were not well tolerated by the subject. The observation that the PCr depletion in soleus was stronger with a flexed knee than with a straight leg, but without depletion reaching the maximum depletion levels of gastrocnemius, has also been reported by Valkovic et al.^20^

Despite the finding that *Q*_max_ was not significantly different between gastrocnemius and soleus, the notion of a metabolic difference between the muscle tissues is supported by the trend towards different pH reached at similarly elevated PCr depletions: PCr depletions above 60 *%* were associated with a pronounced fall in pH for gastrocnemius medialis, which is associated with the lactate threshold and was previously observed with a similar protocol.^4^ Interestingly, however, the pH fall was less pronounced when comparable PCr depletions were reached in soleus. Such a difference could be linked to intrinsic metabolic differences between the muscle tissue of gastrocnemius and soleus. This might reflect the fact that soleus consists of less glycolytic muscle fibres and could be the focus of a future study.

## 5 CONCLUSION

The knee angle influences the distribution of workload between gastrocnemius and soleus muscles during plantar flexion. Consequently, it affects metabolic parameters measured with ^31^P MRS during exercise and recovery. The recruitment of gastrocnemius decreased significantly when increasing the knee angle, which was reflected by a negative linear correlation between knee angle and PCr depletion and by significant changes of end‐exercise pH values and PCr recovery time. The effect in soleus was opposite and less pronounced. The results of this study, based on time‐resolved multivoxel ^31^P MRS during calf muscle exercise, suggest that the knee angle should be considered in the widely used study designs employing localized or unlocalized data acquisition for the investigation of human exercise physiology.
